# Differential Progression of Motor Dysfunction Between Male and Female Fragile X Premutation Carriers Reveals Novel Aspects of Sex-Specific Neural Involvement

**DOI:** 10.3389/fmolb.2020.577246

**Published:** 2021-01-12

**Authors:** Danuta Z. Loesch, Flora Tassone, Anna Atkinson, Paige Stimpson, Nicholas Trost, Dean L. Pountney, Elsdon Storey

**Affiliations:** ^1^Department of Psychology and Counselling, School of Psychology and Public Health, La Trobe University, Bundoora, VIC, Australia; ^2^Department of Biochemistry and Molecular Medicine, School of Medicine, University of California, Davis, Davis, CA, United States; ^3^MIND Institute, University of California Davis Medical Center, Davis, CA, United States; ^4^Wellness and Recovery Centre, Monash Medical Centre, Clayton, VIC, Australia; ^5^Medical Imaging Department, St Vincent's Hospital, University of Melbourne, Parkville, VIC, Australia; ^6^Neurodegeneration Research Group, School of Medical Science, Griffith University, Gold Coast Campus, Southport, NC, Australia; ^7^Department of Medicine (Neuroscience), Monash University, Alfred Hospital Campus, Melbourne, VIC, Australia

**Keywords:** FMR1 premutation, CGG repeat, female carriers, motor scores, progression rates, gender differences

## Abstract

Expansions of the CGG repeat in the non-coding segment of the FMR1 X-linked gene are associated with a variety of phenotypic changes. Large expansions (>200 repeats), which cause a severe neurodevelopmental disorder, the fragile x syndrome (FXS), are transmitted from the mothers carrying smaller, unstable expansions ranging from 55 to 200 repeats, termed the fragile X premutation. Female carriers of this premutation may themselves experience a wide range of clinical problems throughout their lifespan, the most severe being the late onset neurodegenerative condition called “Fragile X-Associated Tremor Ataxia Syndrome” (FXTAS), occurring between 8 and 16% of these carriers. Male premutation carriers, although they do not transmit expanded alleles to their daughters, have a much higher risk (40–50%) of developing FXTAS. Although this disorder is more prevalent and severe in male than female carriers, specific sex differences in clinical manifestations and progress of the FXTAS spectrum have been poorly documented. Here we compare the pattern and rate of progression (per year) in three motor scales including tremor/ataxia (ICARS), tremor (Clinical Tremor Rating scale, CRST), and parkinsonism (UPDRS), and in several cognitive and psychiatric tests scores, between 13 female and 9 male carriers initially having at least one of the motor scores ≥10. Moreover, we document the differences in each of the clinical and cognitive measures between the cross-sectional samples of 21 female and 24 male premutation carriers of comparable ages with FXTAS spectrum disorder (FSD), that is, who manifest one or more features of FXTAS. The results of progression assessment showed that it was more than twice the rate in male than in female carriers for the ICARS-both gait ataxia and kinetic tremor domains and twice as high in males on the CRST scale. In contrast, sex difference was negligible for the rate of progress in UPDRS, and all the cognitive measures. The overall psychiatric pathology score (SCL-90), as well as Anxiety and Obsessive/Compulsive domain scores, showed a significant increase only in the female sample. The pattern of sex differences for progression in motor scores was consistent with the results of comparison between larger, cross-sectional samples of male and female carriers affected with the FSD. These results were in concert with sex-specific distribution of MRI T2 white matter hyperintensities: all males, but no females, showed the middle cerebellar peduncle white matter hyperintensities (MCP sign), although the distribution and severity of these hyperintensities in the other brain regions were not dissimilar between the two sexes. In conclusion, the magnitude and specific pattern of sex differences in manifestations and progression of clinically recorded changes in motor performance and MRI lesion distribution support, on clinical grounds, the possibility of certain sex-limited factor(s) which, beyond the predictable effect of the second, normal FMR1 alleles in female premutation carriers, may have neuroprotective effects, specifically concerning the cerebellar circuitry.

## Introduction

The fragile X premutation, consisting of a small expansion of a CGG repeat ranging from 55 to 200 in the non-coding section of the Fragile X Mental Retardation 1 (*FMR1*) X-linked gene, is associated with the variety of abnormal conditions (Hagerman and Hagerman, 2004). These premutation (PM) alleles are relatively common, with the population prevalence ranging from 1 in 130 to 1 in 250 females, and from 1 in 250 to 1 in 810 males (Hagerman, 2008; Fernandez-Carvajal et al., 2009). The premutation-size unstable CGG repeat may expand into a larger (>200) repeat size from the female carriers to their offspring, causing the severe neurodevelopmental disorder- Fragile X syndrome (FXS) (Loesch and Hagerman, 2011). The most severe premutation-associated condition affecting both male and female carriers of the PM allele is the late- onset progressive neurodegenerative condition termed Fragile X-Associated Tremor/Ataxia Syndrome (FXTAS). This affects 40–50% of males over the age of 55, but only 8–16.5% female carriers in the same age group (Cronister et al., 1991; Rodriguez-Revenga et al., 2009; Loesch and Hagerman, 2011; Hagerman and Hagerman, 2013). The much lower risk of FXTAS in female than in male carriers can be, at least partly, attributed to the mitigating effect of the normal *FMR1* allele on the second X chromosome (Jacquemont et al., 2004).

Traditionally, the standard diagnostic (core) neurological features of FXTAS comprise intention tremor, gait ataxia, and MRI T2 hyperintensities in the middle cerebellar peduncles (MCP sign) (Brunberg et al., 2002; Hagerman and Hagerman, 2016). Additional, non-core features contributing to the diagnosis, include parkinsonism, cognitive decline seen in the later stages of this condition (executive function and episodic memory deficits), neuropathy (Soontarapornchai et al., 2008; Apartis et al., 2012), and other MRI findings, such as global brain atrophy and white matter disease (Jacquemont et al., 2004; Adams et al., 2007; Rodriguez-Revenga et al., 2009; Loesch et al., 2011; Apartis et al., 2012; Wheeler et al., 2014; Hermanson et al., 2015), especially in the splenium of the corpus callosum, and the basis pontis, but also around lateral ventricles and in the deep white matter of brain hemispheres. Typical FXTAS neuropathological changes consist of ubiquitin-positive intranuclear inclusions abundant in neurones and astrocytes (Greco et al., 2002), extending to autonomic nervous and neuroendocrine systems and myocardial cells (Louis et al., 2006; Greco et al., 2007; Hunsaker et al., 2011).

One component of the nuclear inclusions is the *FMR1* mRNA (Tassone et al., 2004), which has previously been found to be elevated in the blood of corresponding to increased CGG repeat number (Tassone et al., 2000). These findings have led to a hypothesized pathogenetic mechanism that involves a toxic gain-of-function of the expanded CGG-repeat mRNA, which arises through the adventitious binding/sequestration by the CGG repeat of one or more proteins, contributing to dysfunction and/or death of the cell (Jin et al., 2003; Polussa et al., 2014). An alternative model for FXTAS pathogenesis has been proposed, in which “toxic” peptides are generated by initiating translation at non-AUG codons located upstream of the CGG-repeat element. It has been shown that this process, known as Repeat-associated non-AUG (RAN) translation, which is proportional to the extent of expanded *FMR1* mRNA elevation, leads to “chimeric” peptide synthesis including a poly-glycine peptide that is toxic to cells. This peptide is detectable in both the intranuclear inclusions of subjects with FXTAS and in the inclusions of the Dutch premutation CGG-repeat mouse model (Todd et al., 2013; Boivin et al., 2018; Glineburg et al., 2018). These and other postulated additional mechanisms, associated with CGG expansions within the premutation range, and leading to the severe neuropathological changes underlying FXTAS, have been reviewed in 2015 (Hagerman and Hagerman, 2015).

The most common premutation-associated condition in females, occurring in ~20% of carriers of this allele, is premature ovarian failure (FXPOI). This term indicates early menopause (defined as occurring before 40 years of age; Hagerman and Hagerman, 2013). However, the effect of the PM alleles may extend beyond those two definitive disorders, FXTAS and FXPOI, especially in the female carriers, where other physical changes, such as autoimmune thyroid disease and other immune-related disorders, as well as psychiatric problems including social phobia, hostility, obsessive/compulsive behavior, and anxiety/depression can be seen; in addition, common disorders such as fibromyalgia, hypertension and migraines have been reported to be more common in female carriers that in the general population (Coffey et al., 2008; Soontarapornchai et al., 2008; Bourgeois et al., 2009, 2011; Roberts et al., 2009; Leehey et al., 2011; Loesch and Hagerman, 2011; Seltzer et al., 2012; Winarni et al., 2012; Au et al., 2013; Wheeler et al., 2014). However, most of the data obtained by comparing samples of carriers with non-carriers have yielded inconsistent results, in part attributable to selection bias (Hunter et al., 2008; Allen et al., 2020), and thus provide limited insight into the effects of PM alleles on female phenotypes.

There have been fewer studies exploring subtle impairments that might be directly related to pre-symptomatic brain changes in either male or female PM carriers, in the absence of overt neurological symptoms or signs. In males, the studies based on MRI analyses revealed subtle changes in the integrity of white matter without, or prior to, the occurrence of FXTAS (Wang et al., 2012, 2013; Battistella et al., 2013), and abnormal trajectories of cerebellar and brain stem volume loss from early adulthood (Wang et al., 2017). In female carriers, the findings of deficits on a range of tasks of executive functioning requiring rapid temporal responses (Shelton et al., 2016), or of subtle impairment of postural stability (O'Keeffe et al., 2019), compared with control non-carriers, suggested that there may be a slow but measurable decline in executive functioning and/or degradation of motor control in apparently asymptomatic individuals. The presence of intranuclear inclusions typical of FXTAS has been reported in both FXTAS and non-FXTAS female carriers (Tassone et al., 2012), and reinforces evidence for an accumulation of subclinical pathological processes over a broad age range associated with PM carriage. However, the progression and outcome of these sub-symptomatic changes in older age, including the core FXTAS manifestations of tremor, gait ataxia and the MCP sign, have not yet been addressed. Moreover, despite the large volume of studies to identify possible physical and mental health problems in female PM carriers, there is still insufficient understanding of the effect of skewed X-inactivation on the phenotypic changes and their trajectory in these carriers. This difficulty can be attributed to the well documented brain-blood difference in *FMR1* mRNA expression, and potentially of the CGG expansion (Tassone et al., 2004; Pretto et al., 2014a,b; Zhao et al., 2019). This reflects the absence of a relationship between epigenetic measure (activation ratio, AR) and clinical phenotypes, which has been commonly observed in PM females in a number of the relevant studies, reviewed in: Jiraanont et al. (2017). These authors acknowledge the complexity of the mechanisms linking the PM allele to female clinical phenotypes, and they suggest the existence of other still unidentified factors impacting on these phenotypes.

In this study we aimed to test the hypothesis that subtle phenotypic changes, once initiated, continue to progress, but generally at a much slower and less uniform rate in female than in male PM carriers. Our second aim was to delineate the pattern of sex differences in clinical motor, cognitive, and psychiatric involvement in those carriers classified as Fragile X Spectrum Disorder (FSD), that is, manifesting at least one major or two minor features consistent with FXTAS. The relationships of severity and progression of phenotypic scores with the CGG repeat size were also assessed for each sex separately. These results, combined with evidence from relevant MRI FLAIR images showing sex differences in a topography of white matter lesions, suggest the existence of specific factors that may alleviate the severity and progress of particular neurological manifestations in female PM carriers.

## Methods

### Subjects

**Figure 1:**
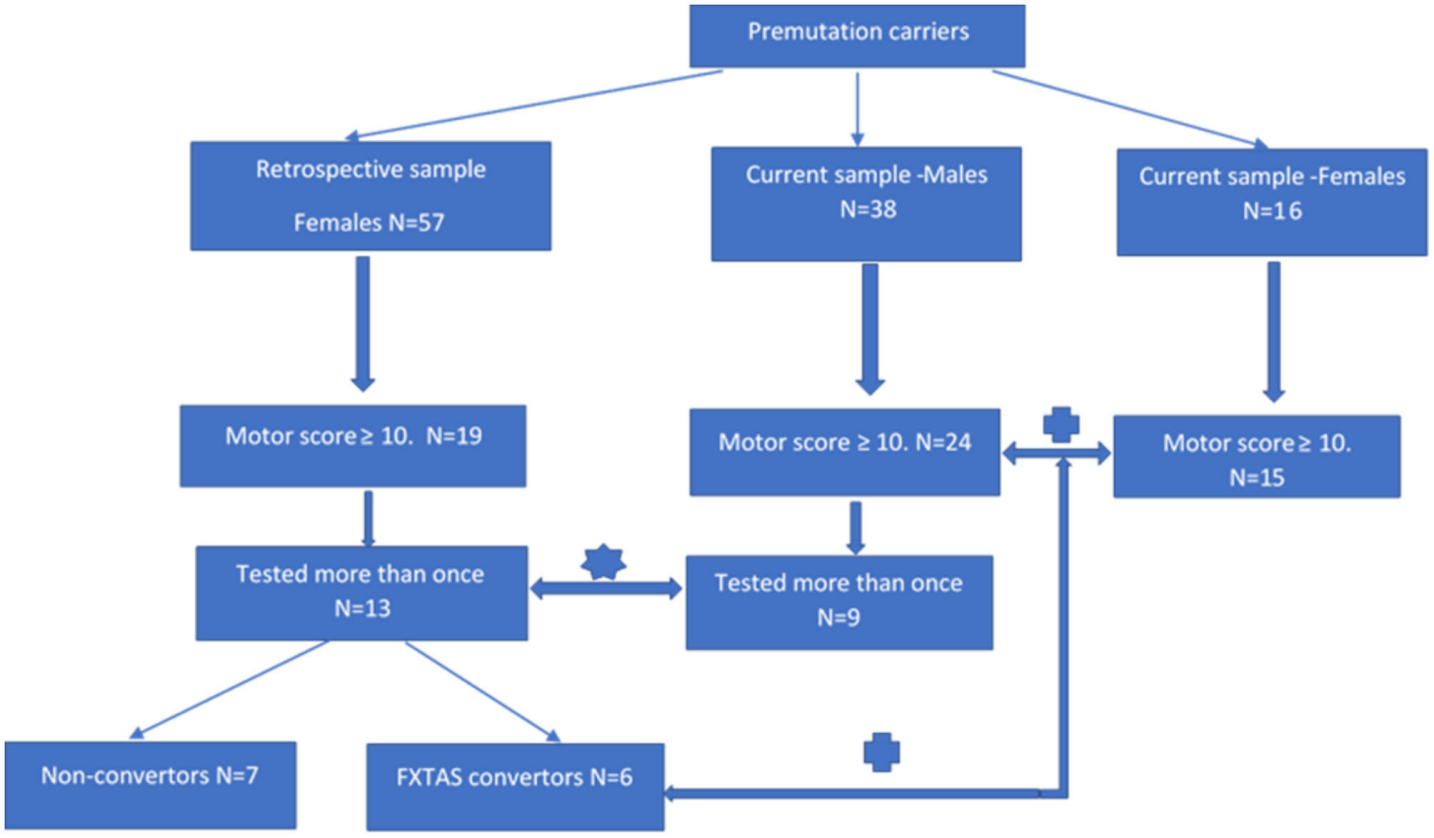
Flowchart showing individual steps of recruitment of the participants of progression and cross-sectional aspects of analysis. 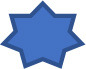 rate of progression study; 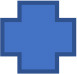 male-female comparison study.

There were two aspects of this study that require two different (but overlapping) sources of participants, all of whom were adults (>50 years of age) carrying the PM allele. The first source of both male and female participants was a major research project continuing from 2012 at La Trobe University and supported by the National Institutes of Health, USA. This project's male and female participants (later termed “*current”* sample as they are still being studied in a longitudinal fashion) were originally recruited through fragile X families' admissions to the Victorian Genetic Counselling Clinic of the Murdoch Institute, or referred from several neurology clinics associated with the University of Melbourne and Monash University; the minority (some residing in the other states) were self-referred by postings in the community through The Australian Fragile X Association. The second source of female participants (later termed the “*retrospective”* sample) was our earlier 2008–2010 project supported by a research grant from the National Health and Medical Research of Australia (NHMRC) to ES and DZL. These females had originally been ascertained either through their FXS children diagnosed at the Genetic Counselling clinics in Victoria and South Australia, or were more distant relatives of these probands identified through cascade testing. Except for one Asian (Chinese) male and one (Thai) female, all participants were white Caucasians. The two-*current* and *retrospective -*samples were used in both progression rate and male-female comparisons aspects of this study, using the inclusion criteria specified below in section “Cross-Sectional Male-Female Comparisons”, and in a flowchart (Figure 1), where sample sizes at every step of sample selection are also provided.

#### Progression Rate

The sample of 13 females aged 37–66 years has been selected by retrospective inspection of the results of motor scale scores from the sample of 57 adult females carrying the premutation allele, who underwent comprehensive neurological testing between 2008 and 2010, conducted by two specialist neurologists (ES and DZL-authors of this manuscript). In this same study (supported by the NHMRC project grant), these females also underwent a battery of neuropsychological and psychiatric pathology tests under the supervision of ES, using standard protocols. Partial retrospective analysis of SCL-90 data collected in this sample was described in our 2015 publication (Loesch et al., 2015). Our inspection of the raw data from this earlier (“*retrospective*”) sample revealed that, amongst apparently asymptomatic carriers, 19 individuals scored ≥10 on the ICARS (2 SD above the mean) and/or ≥10 on the CRST (1 SD above average), and we decided to re-test these individuals after a 9–10 years interval. Thirteen of the 19 females from this subgroup (aged 36–66) were available for the follow-up testing within the present progression study (two declined, two have died of unknown cause, and two were not contactable). The follow-up 2019 assessment comprised, as far as possible, the same battery that had been used in the first run of testing in 2009. Based on the results of the follow-up assessment, six of these females met our criteria for the category of FSD (converters from the “*retrospective”* group in Figure 1), and they were also combined with 15 female carriers from the “*current*” group for the second part of this study aimed at comparing the phenotypic scores between male and female carriers classified as affected category.

The sample of nine male PM carriers aged 50–72 years, was used to compare with the data on the rate of progression obtained from the female sample. These individuals were selected from amongst 38 PM carriers who have been undergoing routine testing within our major NIH 2012–2021 longitudinal *(“current”*) study. In order to match the criteria of inclusion between the two sexes as close as possible, we only selected those (nine) males from this total sample who have been tested at least twice and, like female participants, had at least one (ICARS or CRST) motor score ≥10 on initial testing. The remaining differences between male and female samples, which could have potentially biased the results of comparison, were accounted for as described in “Statistical Analysis” and “Results” sections.

#### Cross-Sectional Male-Female Comparisons

The total sample of 24 male participants aged 48–80, and 21 female participants aged 37–78 were included in the second part of this analysis, which compared all phenotypic scores between the male and female carriers classified as having FSD, and displayed elevation of at least one motor score ≥10 (complying with the criterion applied to the 13 females from the “*retrospective”* sample). All male individuals were participants in the ongoing NIH-supported project (“*current*” sample). Six females were selected from the “*retrospective*” group (as described above), and 15 -from the “*current”* group. Within this group, the average follow-up period was 5.00 (2–7) years for males and 10.15 (9–11) years for females. The CGG repeat size averaged 85.1 in males and 80.2 in females; the difference in average repeat length of five repeats is expected to be negligible considering test error of ± 2 repeats and the PM range of 55–200.

### Testing Protocols

#### Neurological and Cognitive Measures

Two neurologists specializing in movement disorders (ES and DZL), with relevant experience in these scales from previous studies, administered the three motor rating scales. These scales consisted of the Unified Parkinson's Disease Rating Scale Part III-Motor (UPDRS-III) (Fahn et al., 1987), the International Cooperative Ataxia Rating Scale (ICARS) (Trouillas et al., 1997), and the Clinical Rating Scale for Tremor (CRST) (Fahn et al., 1993), the first two of which have established inter-rater reliabilities (Richards et al., 1994; Storey et al., 2004; Stacy et al., 2007). The Vocabulary and Matrix Reasoning subtests of the Wechsler Adult Intelligence Scale (Third Edition; WAIS-III) were used to calculate a prorated Full-Scale IQ score (Wechsler, 1997), with Matrix Reasoning providing a measure of non-verbal reasoning. WAIS-III Digit Spans (forward and backward separately) were employed as measures of attention and working memory, respectively (Wechsler, 1997).

#### Psychiatric Pathology Test Scores

The SCL-90-R (Derogatis, 1994)—a 90 item self-administered questionnaire was chosen as an instrument that can efficiently provide information on a broad range of relevant symptom clusters and psychological concerns. It has good validity and reliability (test-retest: 0.80–0.90). Each of the 90- items is rated on a five-point Likert scale of distress, ranging from “not at all” (0) to “extremely.” The subject is asked to respond to questions based on how much a given problem has “distressed or bothered” him or her during the past week, including the present day. It is typically completed in about 12–15 min. Here we report the data on a summary score providing a measure of overall psychological distress—the Global Severity Index (GSI), and on nine primary symptom dimensions: Somatization, Obsessive-compulsive, Interpersonal Sensitivity, Depression, Anxiety, Hostility, Phobic Anxiety, Paranoid Ideation, and Psychoticism.

#### Genetic Molecular Measures

##### CGG Sizing

Genomic DNA was isolated from peripheral blood lymphocytes using standard methods (Purygene Kit; Gentra, Inc., Minneapolis, MN). For Southern blot analysis, 10 mg of isolated DNA were digested with EcoRI and NruI. Hybridization was performed using the specific *FMR1* genomic dig-labeled StB12.3 probe as previously described (Tassone et al., 2008). Genomic DNA was also amplified by PCR (Filipovic-Sadic et al., 2010) DNA analysis was performed in the Laboratory of Dr. Tassone at the MIND Institute, UC Davis.

##### FMR1 mRNA Expression Level Measurements

This assay was also conducted at the MIND Institute. Total RNA was isolated from 3 mL of blood collected in Tempus tubes (Applied Biosystems, Foster City, California, USA). The measurement of *FMR1* mRNA expression levels was carried out by quantitative Real-Time qRT-PCR using custom-designed Taqman gene expression assays (Applied Biosystems) as previously described (Tassone et al., 2000).

##### Activation Ratio

Activation ratio (AR) indicates the proportion of cells that carry the normal allele on the active X chromosome, so that AR = 1.00 indicates a normal allele active in 100% of the cells. It was measured based on the intensity of the appropriate bands on Southern blots as described in Tassone et al. (1999).

### Statistical Analysis

Data were analyzed using the Statistical Package for the Social Sciences version 26 (SPSS; IBM Corporation; Armonk, NY, USA). Summary statistics for sample characteristics, cognitive, psychiatric, and motor scores are presented as means and standard deviations (SD). Gender differences in parameters, and progression per year, were analyzed using independent sample *t*-tests. To account for inter-individual differences in baseline scores, progression change for each of the parameters was calculated as the percentage change between baseline (T1) and follow up (T2), with the following formula: Percentage Progression per year = [(T2 score – T1 score)/T1 score ^*^ 100]/ years between sessions. Paired sample *t*-tests were conducted to examine progression between Time 1 and Time 2 for each sex. There was no significant age difference between the sexes, and therefore age was not adjusted for in any analyses: for T1: *P* = 0.054, 95% CI −18.4, 0.19; for T2: *P* = 0.375, 95% CI −13.0, 5.13. (both *t*-test for unequal variances). Neither was there any significant age differences between the sexes for the cross-sectional component of the study (see Table 2), so correction for age was, again, unnecessary. The relationship between each of the phenotypic scores, and percentage progression per year (as outcome), and CGG repeat size and *FMR1* mRNA, was assessed using linear regression.

## Results

In this study, we considered the threshold for inclusion to be a score of ≥10 for the ICARS, which is 2 SD above average (4.07, SD 2.19) (Fitzpatrick et al., 2012). We are unaware of any relevant published normative values for the CRST, but used a sensitive cut off ≥10, being 1 SD above average in the control group of non-carrier males from our previous study (6.1, SD 4.85) (Loesch et al., 2005). Abnormality on UPDRS was signified by a score of ≥ 7, being two SD above average (1.9 ± 2.0) (Postuma et al., 2012). However, given that parkinsonism is not the major feature of FXTAS and is relatively common in the general population, the UPDRS score was used purely for analysis. Among 19 carriers who were selected based on these criteria, four presented with an elevation of ICARS score only, seven showed elevation of both ICARS and CRST scores, one presented with an elevation of UPDRS score in addition to elevation of their ICARS score, and seven with elevation of only CRST.

### Progression Rate

Thirteen female carriers were available for the progression component of the study. Descriptive statistics of this group, including at baseline (T1) and at the follow-up assessment (T2) on the motor scales and cognitive and psychiatric pathology scores, are given in Table 1B. The significance of progression rate in these scores between times T1 and T2 (*p*-values from the *t*-test and confidence intervals, CI), are also given. In addition, the values of effect sizes are also reported. The corresponding parameters are also presented in Table 1A, for nine males selected from a larger- *current*-sample of PM carriers using the same motor scale criteria. Although the time length between the two assessments was typically shorter for males than for the female sample, it is evident that the T2-T1 differences are consistently greater in the males. This difference is much greater than should be expected in females assuming random X-inactivation. This especially applies to ICARS-gait and stance- domains, and CRST, where these differences are three times higher in males than in females, not reaching significance in the latter. This is in contrast with the ICARS (kinetic) domain, where the T2-T1 difference in males is less than twice that of the female value. The same applies to the UPDRS, where the progress is relatively small in either sex, without reaching statistical significance in the male sample. Although the progress assessed by T2-T1 differences in cognitive scores, including the Pro-Rated IQ and the Wechsler subtests, was negligible in either sex, it was evident (and highly significant) for the SCL90 GSI (summary) score.

**Table 1:** Summary statistics for all phenotypic scores at Time 1 and Time 2 in male (A) and female (B) samples included in the progression analysis, and the *p* values for significance of the T2-T1 differences for each sex separately.

	**Paired differences**									
**Variable**	***N***	**T1 Mean**	**SD**	***N***	**T2 Mean**	**SD**	**Mean diff. [95% CI]**	**SEM**	***P*** **value**	**Effect size (*****g*****)**
**A**
Age	9	61.6	7.8	9	66.6	7.7	5 [3.7, 6.3]	0.56	N/A	N/A
UPDRS	9	9.8	10.0	9	17.3	13.7	7.6 [0.5, 15.6]	3.5	0.062	0.57
CRST	7	28.6	24.8	9	40.4	30.1	11.3 [5.3, 17.3]	2.4	**0.004**	0.37
ICARS total	9	17.9	11.8	9	28.7	13.5	10.8 [6.7, 14.9]	1.8	**0.000**	0.77
ICARS gait	9	4.6	3.0	9	8.3	4.1	3.8 [1.6, 5.9]	0.94	**0.004**	0.94
ICARS kinetic	9	11.3	7.3	9	17	7.2	5.7 [3.9, 7.4]	0.76	**0.000**	0.70
Vocab SS	9	9.2	3.3	9	10.2	3.2	1 [−0.3, 3.3]	0.58	0.122	0.28
MR SS	7	12.0	2.0	9	11.7	3.2	−1.4 [−3.9, 1]	0.99	0.202	0.52
DS Forwards	8	9.5	3.0	9	9.8	2.7	0 [−2.3, 2.3]	0.96	1.000	0.40
DS Backwards	8	6.8	2.1	9	5.9	1.4	−0.9 [−2.7, 1]	0.79	0.304	0.43
Pro-rated IQ	9	102.1	12.0	9	106.1	15.9	4 [−4.7, 12.7]	3.8	0.318	0.26
SCL-90 GSI	6	58.3	19.6	9	55.7	13.8	−2.7 [−19.6, 14.3]	6.6	0.703	0.13
**B**										
Age	13	52.5	13.0	13	62.6	12.7	10.1 [9.7, 10.6]	0.22	N/A	N/A
UPDRS	13	4.5	4.5	13	8.7	4.9	4.1 [1.1 ,7]	1.34	**0.011**	0.78
CRST	13	12.7	5.6	13	16.3	7.1	3.6 [−2, 9.3]	2.60	0.189	0.53
ICARS total	13	10.1	4.2	13	15.7	6.2	5.8 [3.4, 8.3]	1.13	**0.000**	0.99
ICARS gait	13	3.6	1.9	13	4.9	2.2	1.3 [0.48, 2.1]	0.38	**0.005**	0.59
ICARS kinetic	13	5.8	2.7	13	9.1	3.9	3.2 [1.45, 5]	0.82	**0.002**	0.91
Vocab SS	12	8.3	2.8	11	10	1.5	1.1 [0.07, 2.1]	0.46	0.038^*^	0.54
MR SS	13	9.4	3.5	12	8.9	3.3	−1.1 [−3.4, 1.2]	1.1	0.327	0.32
DS Forwards	12	9.3	1.9	12	9.5	2.1	0.09 [−0.97, 1.2]	0.48	0.852	0.05
DS Backwards	12	5.8	1.9	12	5.3	1.0	−0.6 [−1.8, 0.54]	0.52	0.255	0.39
Pro-rated IQ	12	96.8	7.5	11	97.6	7.1	0.73 [−5.7, 7.1]	2.88	0.806	0.09
SCL-90 GSI	13	52.1	9.8	11	57	2.8	4.9 [1.7, 8.2]	1.46	**0.007**	0.48

*All males came from the current sample, all females came from the retrospective sample*.

*Vocab SS, vocabulary scaled score from WAIS-3; MR SS, matrix reasoning from WAIS-3; DS forwards, digit span raw scores separately from WAIS-3. P values are for the two-sided t-test*.

*Figures in bold were statistically significant at p < 0.05*.

* *T2-T1 difference is positive. p values are for the two-sided paired t-test; CI, confidence intervals*.

**Figure 2:**
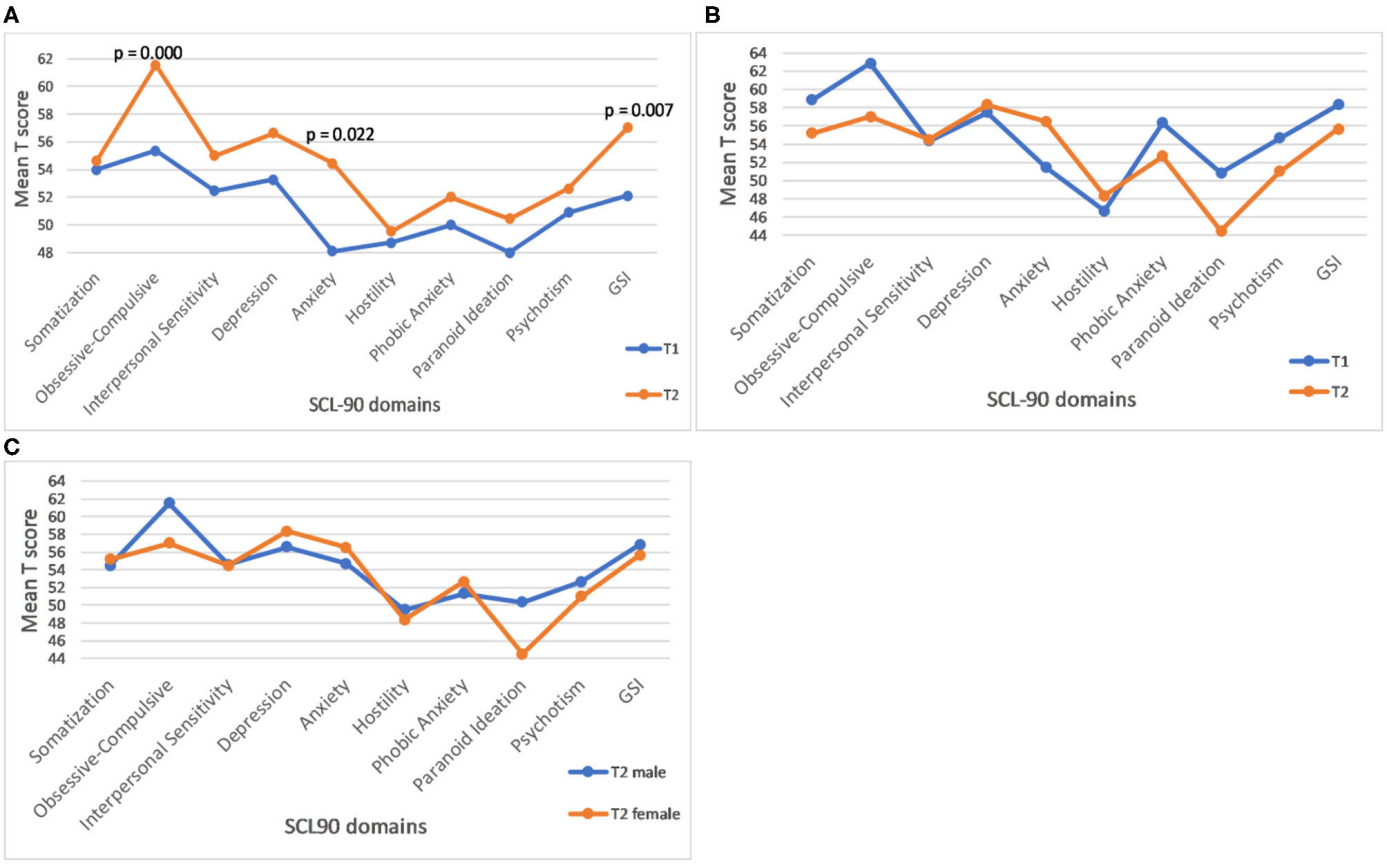
Profile plot of mean baseline and follow-up of SCL-90 in female carriers. **(A)** The *p* values represent significance of the rate of progression. **(B)** Profile plot of mean baseline and follow-up of SCL-90 in male carriers. **(C)** Gender differences in the rate of progression of SCL-90 GSI score and individual domains in male and female carriers. **(C)** None of the male-female differences is statistically significant.

The results of the follow-up assessments for the SCL90 GSI (summary) score, as well as SCL-90 profiles based on all 9 domains in female and male samples, respectively are illustrated in Figure 2. The rate of increase per year is significant for the GSI score (Figure 2 and Table 1), as well as for Anxiety and Obsessive-Compulsive domains, in females (Figure 2A) but not in males (Figure 2B). Sex differences for the rate of increase for these (Figure 2C), and for all other SCL 90 domains or GSI scores, were not statistically significant.

**Figure 3:**
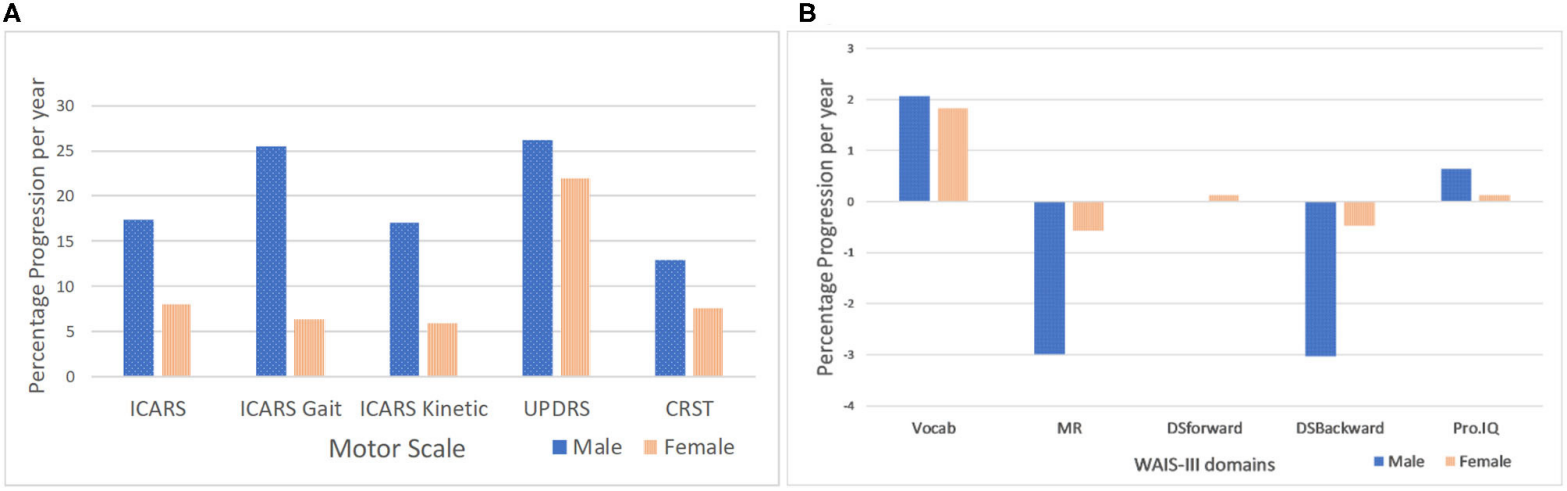
Male-female differences in the rate of progression per year in motor scores adjusted for T1 scores. **(A)** *P* values are for statistically significant differences between males and females for rate of progression (*t-test for unequal variances). *P* values for T2-T1 measures (male-female differences) adjusted for T1 scores: ICARS total (*p* = 0.029); ICARS Gait (*p* = 0.013); ICARS Kinetic (*p* = 0.026*); CSRT and UPDRS (*p* > 0.05)- *P* values for T2-T1 measures (male-female differences) not adjusted for T1 scores: ICARS total (*p* = 0.003*); ICARS Gait (*p* = 0.02*); ICARS Kinetic (*p* = 0.008*); and CSRT (*p* = 0.000*); UPDRS (*p* > 0.05). Male-female differences in the rate of progression per year in WAIS-III scores adjusted for T1 scores. **(B)** Males did not progress significantly in any Wechsler subscale scores or in Prorated IQ between T1 and T2. Females only showed (marginally) significant improvement in the Vocabulary subscale (*p* = 0.038).

The magnitude and pattern of progression for all motor and cognitive measures are displayed in Figures 3A,B, respectively, including the *p*-values for male-female differences in progression rates. Since there was no significant male-female difference in age in either T1 (baseline) or T2, no age correction was applied. However, in order to allow comparison between males and females, the T2-T1 difference for each individual measure was divided by the number of years between the two assessments, to yield annual progression rates. Moreover, when assessing sex differences in the progression of motor and cognitive impairment, we also expressed measures of progression for each sex as a proportion of baseline (T1) value, to correct for possible baseline severity bias caused by differences between male and female samples. The *p* values from the *t*-tests representing significance of gender differences for the rate of progression, given in the Legend to Figure 3, are given both, with and without adjustment for baseline (T1) scores, respectively. The outcome was similar for these two models, with the exception of the CRST score, where male-female difference was significant only for the model without T1 adjustment, reflecting the possibility of T1-related bias. This, apart from a limited accuracy of the *t*-test in small samples, explains rare inconsistencies between the estimates of *p* values given in this figure and in Table 2, respectively.

**Table 2:** Cross sectional study.

**Variable**	**Males**			**Females**			**Gender differences**			
	**N**	**Mean**	**SD**	**N**	**Mean**	**SD**	**Mean diff. [95% CI]**	**SE**	***P* value**	**Effect size (*d*)**
Age	24	64.3	8.1	21	63.1	10.9	−1.19 [−6.9, 2.8]	2.84	0.667	0.12
CGG repeats	23	85.1	20.2	18	76.6^a^	18.6	−8.5 [−20.9, 3.9]	6.13	0.173	0.43^b^
RNA level	16	2.46	1.1	13	1.24	0.5	−1.22 [−1.9, −0.6]	0.3	**0.001**	1.33^b^
UPDRS	23	14.3	13.2	21	9.6	6.8	−4.77 [−11.1, 1.6]	3.12	0.135^*^	0.46
CRST	24	30.4	25.3	21	17.5	7.5	−12.89 [−24.0, −1.8]	5.42	0.025^*^	0.69
ICARS total	24	24.5	13.9	21	16.95	7.2	−7.6 [−14.1, −0.99]	3.25	0.025^*^	0.68
ICARS gait	24	7.9	5.0	21	5.0	3.0	−2.87 [−5.3, −0.4]	1.21	0.023^*^	0.70
ICARS kinetic	24	14.0	7.5	21	10.4	4.6	−3.62 [−7.3, 0.08]	1.82	0.055^*^	0.58
Vocab SS	23	10.1	2.9	19	11.1	2.3	0.97 [−0.7, 2.6]	0.83	0.245	0.36^b^
MR SS	23	10.6	4.0	20	10.3	3.5	−0.32 [−2.6, 2.0]	1.16	0.787	0.08
DS Forwards	23	9.7	2.4	20	9.6	2.0	−0.15 [−1.5, 1.2]	0.68	0.832	0.07
DS Backwards	23	5.8	1.6	20	5.6	0.9	−0.23 [−1.1, 0.6]	0.42	0.590	0.17
Pro-rated IQ	23	101.7	19.3	19	104.5	12.6	2.73 [−7.3, 12.8]	4.95	0.584^*^	0.16^b^
SCL90 GSI	15	56.2	13.0	18	56.8	7.9	0.56 [−7.1, 8.2]	3.65	0.881	0.05^b^

*Summary statistics for genetic and phenotypic scores in FSD males and females, and between-group differences. All male data come from the current sample, female data comes from the current (15) and the retrospective (6) samples*.

a *The values for two premutation/full mutation female mosaics excluded from this estimate*.

b *Hedges' g*.

* *t-test for unequal variances. Figures in bold were statistically significant at p < 0.05*.

The data presented in Figure 3 show that sex differences for the rate of score increases are most prominent (and highly significant) for the ICARS scale. The rate for the total ICARS in males is more than twice that in females; for the ICARS Kinetic domain the rate in males is nearly three times of that in females; and for the ICARS Gait domain- nearly four time greater in males than in females. In contrast, the male-female difference in the rate of increase is much smaller (and insignificant) on the CRST, and negligible on the UPDRS. There is noticeable decline, in males only, on two Wechsler subtests- MR and DS Backward (compared with negligible change in the females), but this difference is not significant, reflecting the small samples available.

### Cross-Sectional Male-Female Comparisons

Summary statistics, and the results of comparison of the motor and cognitive scale scores between cross-sectional samples of male and female carriers with FSD, including effect sizes, are shown in Table 2. There are no significant differences either in the age of the participants or in the size of the premutation allele, which falls within the range known to be associated with the highest risk for the occurrence of FSD in either sex. Comparison between the three motor scales revealed significant male-female differences for the ICARS (with a slightly greater size effect for Kinetic than Gait domains) and CRST scores, but not between UPDRS scores. There were no significant differences between male and female samples in cognitive test scores, with the trend toward slightly higher Prorated IQ score in the female than in the male sample (likely accounted for by somewhat higher Vocabulary test scores in the former); notably, the Prorated IQ remained within the population mean in both carrier samples. The differences either in the overall SCL-90 GSI (Table 2) or in any individual SCL-90 domains (data not shown) are insignificant.

### Genotype-Phenotype Relationships

**Figure 4:**
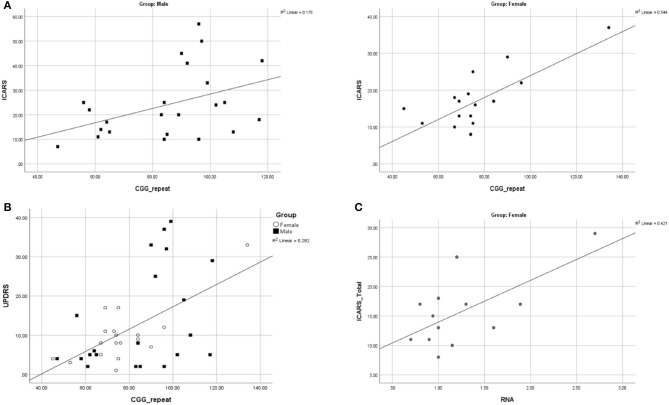
**(A)** Scatterplots illustrating significant relationships between CGG repeat size and ICARS Total in males and females. **(B)** Scatterplot illustrating a significant relationship between CGG repeat size and UPDRS in males and females combined. **(C)** Scatterplots illustrating significant relationships between *FMR1* mRNA and ICARS total in females.

We assessed the regression of the motor and cognitive scores, listed in Table 2, on the number of CGG repeats, in male and female samples. The ICARS total score was the only motor scale showing a significant relationship with the CGG repeat size in both sexes: in the male sample, the correlation coefficient was 0.420, *p* = 0.046; in the female sample, it was 0.737, *p* = 0.000 (scatterplots shown in Figure 4A). These relationships remained significant for both gait (*p* = 0.003) and kinetic (*p* = 0.002) domains in females, but not in males. UPDRS scores were significantly correlated with CGG repeat size in both males (0.435, *p* = 0.043) and females (0.747, *p* = 0.000). Since there was no statistically significant difference in the UPDRS scores between males and females, we conducted this analysis in the combined sample, resulting in a highly significant regression of UPDRS score on CGG repeat size, with the correlation coefficient of 0.531, and *p* = 0.000 (Figure 4B). None of the other motor dysfunction or cognitive decline measures showed significant correlations with the CGG repeat size, with correlation coefficients not exceeding ~0.250. The interpretation of the results of correlation analysis between the motor and cognitive scores and the *FMR1* mRNA levels is limited because of small sample sizes. Nevertheless, the only significant correlation encountered in the female sample was for the ICARS, with correlation coefficient of 0.649, *p* = 0.016, for the total score (Figure 4C), and 0.633, *p* = 0.020, for the Kinetic domain score. There was no relationship between any phenotypic scores and the (highly variable) activation ratio (AR), which averaged 0.57, SD = 0.33, and was assessed only in a sample of 13 females included in progression analysis.

### Magnetic Resonance Imaging Data

**Figure 5:**
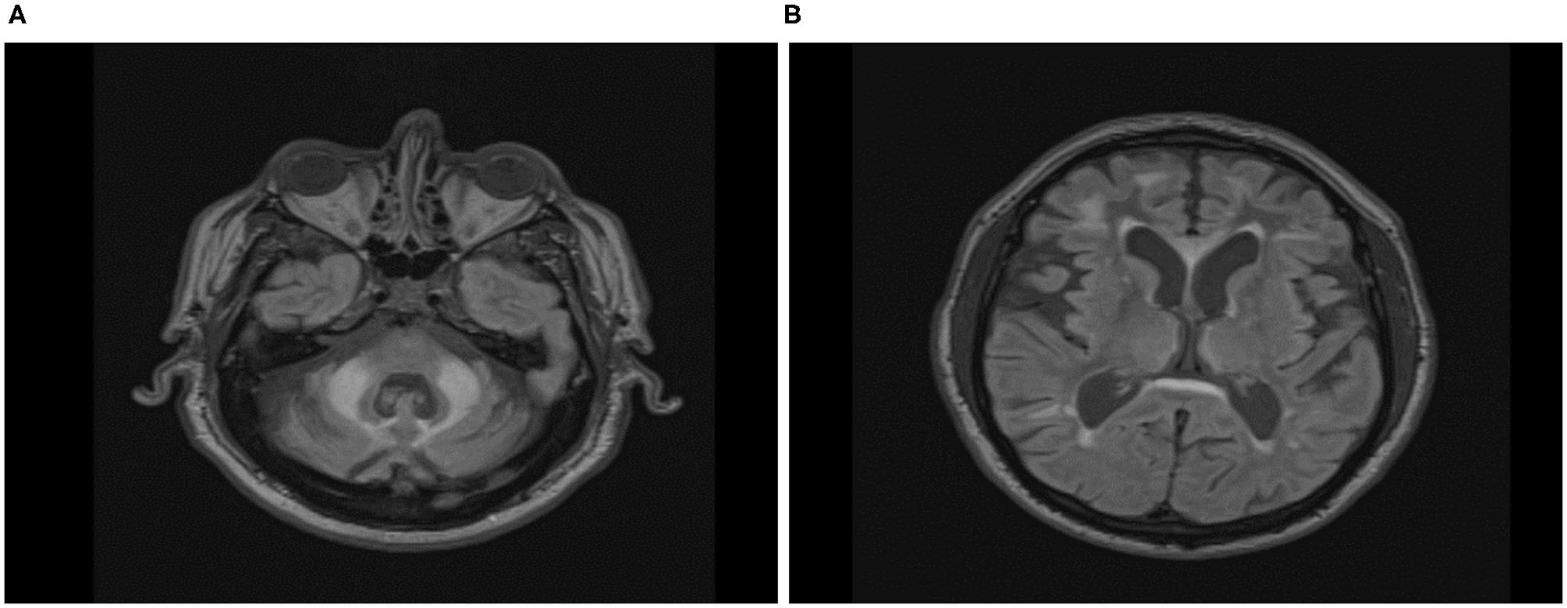
Axial Fluid Attenuated Inversion Recovery (FLAIR) Magnetic Resonance Imaging in FXTAS male **(A)** and female **(B)** premutation carriers. **(A)** Seventy-five-year-old male participant, carrier of 58 CGG repeats, with overt features of FXTAS. ICARS = 22/100; UPDRS = 4/108; CRST:20/156; Prorated IQ = 103. *Wmhs* scattered in both hemispheres, mainly in the frontal region, thick periventricular and splenial bands, and bilateral MCP sign. **(B)** Sixty-seven-year-old female participant, carrier of 73 CGG repeats, with overt features of FXTAS and fibromyalgia. ICARS = 19/100; UPDRS = 11/108; CRST:14/156; Prorated IQ = 84. Few small *wmhs* scattered in both hemispheres, thick periventricular and splenial bands, but absence of *whms* in middle cerebellar peduncles.

The visual examination of T2-Flair MR images of the location of the *wmhs* available to us from 16 male and 12 female carriers included in this cross-sectional analysis revealed the MCP sign in all the males but none of the females, while the *wmhs* in the splenium occurred at the same rate in both males or females. Likewise, the *wmhs* changes within deep hemispheric and periventricular locations were not dissimilar between males and females, though less prominent in the latter. The discrepancy in these manifestations between males and females in the context of the clinical status is exemplified by two of the FXTAS patients included in Table 2, whose illustrative MRI FLAIR images are presented in Figures 5A,B, respectively.

## Discussion

This is the first study to explore male-female differences in manifestions and progression of both the major and minor (including sub-symptomatic) phenotypic changes relevant to FSD, using a quantitative approach. We assessed the rate of progression, over a period of 9–10 years, in all three motor scores (ICARS, UPDRS, and CRST), in several Wechsler cognitive test subscores, and in aspects of psychiatric status, in female PM carriers who were sub-symptomatic at the first examination, but who nevertheless showed an elevation in either the ICARS or the CRST, at baseline. Of those sub-symptomatic females for whom longitudinal data was available (13), half (6) converted to a symptomatic form, with half of these (3) presenting a single feature occuring in FXTAS, and the other half-with diagnosable FXTAS. Analysis of the numerical data from the motor scales showed significant progression for both ICARS and UPDRS scores. Significant progression in all but one (UPDRS) motor scores was also encountered in male carriers selected using the same criteria as for the females. In order to account for the difference between male and female samples in the interval between the two assessments, we considered the rate of progress in relation to the length of this interval in each individual. There was no deterioration in any cognitive score in females, compared with obvious decline (though not significant in this small sample) in the MS, and DS Backward Wechsler subtests in males, both these tests being reliant on various aspects of executive function. However, females demonstrated significant deterioration in psychiatric pathology shown by an increase in the overall SCL90 scale score, and, more specifically, in the Obsessive-Compulsive and Anxiety domain scores, which reflect the prevalent changes reported in cross-sectional surveys (Bourgeois et al., 2009, 2011; Schneider et al., 2016). In contrast, male carriers did not show significant progression either in the SCL90 overall, or in any of its specific domains. Clearly, the significant progression in psychiatric symptoms- especially the anxiety observed in our female carriers-may have important clinical implications by drawing attention to the need for preventative measures, such as psychological intervention, as they age. The only other longitudinal study of anxiety in adult female carriers, although it applied different tools (DSM-IV-I) in a sample of younger females tested 3 years apart, showed concordant results for anxiety, and led the authors to emphasize the importance of attending to psychiatric health in fragile X families (Roberts et al., 2016).

Our data on progression of the premutation-associated changes from sub-symptomatic to clinical forms, especially in females, may have an important impact on future research, as well as on clinical approaches, because it introduces the concept of continuity. Combined with earlier evidence for the existence of subclinical neural or metabolic changes in both male and female carriers over a broad age range (Tassone et al., 2012; Wang et al., 2012, 2013, 2017; Battistella et al., 2013; Shelton et al., 2016; Loesch et al., 2017, 2018; Hocking et al., 2019; O'Keeffe et al., 2019), these data have shown that the focus of such research should be on trajectories rather than on the final outcome of the premutation-associated process. The most recent study, based on a wide range of metabolic and proteomic biomarkers, showed a decline of mitochondrial activities in pre-symptomatic female PM carriers, leading the authors to the conclusion that the development of neurodegeneration or other clinical symptoms in older carriers could be linked to a lifetime accumulation of cellular damage, aggravated by the aging process (Napoli et al., 2020).

The notion of “FXTAS spectrum” (Loesch and Hagerman, 2011; Hagerman and Hagerman, 2013; Roberts et al., 2016), here termed FSD, encompassing manifestations insufficient to formally diagnose FXTAS, and which could simply represent “FXTAS in the making,” has been the first step in this direction. The utility of current strict diagnostic categories for FXTAS is even more questionable considering that one-third of the 19 apparently asymptomatic female carriers in our retrospective sample had significant elevation in at least one motor score, with further marked elevation of these scores over time, evolving into FXTAS-like overt manifestations in over one-third of the “at risk” group over a period of 9–10 years. Notably, in another study, nearly half of premutation females were not aware of having tremor, as shown by CATSYS results (Juncos et al., 2011). Long-term follow-up studies of sub-symptomatic and monosymptomatic carriers would allow determination of the true proportion of subjects in whom elevated motor scores could be considered prodromal of syndromic FXTAS, or in whom the subclinical manifestations become symptomatic.

The major thrust of this study concerned differences between male and female carriers in the trajectory of measurable phenotypic changes. The two earlier studies applied motor scales in both male and female carriers, but these scores were compared, for each sex, between the carriers and control non-carriers. One of these studies, based on a small sample of affected carriers aged over 50, found significant differences in all three (ICARS, UPDRS, and CRST) scores between male, but not female, carriers compared with normal controls (Berry-Kravis et al., 2003). Similar results were obtained in another study using a much larger sample of premutation carriers recruited regardless of their neurological status and employing The FXTAS Rating Scale (a compilation of items from the three standard motor scales). While these scores were significantly worse in the entire group of male carriers compared with non-carrier controls, there was only a trend toward a difference in these scores between female carriers and controls (Leehey et al., 2008). These earlier results gave rise to the notion that there might be other sex-related protective effects on the female FSD phenotype besides the second X chromosome (Leehey et al., 2008). Although neither of these findings can be compared with our data, especially as direct comparisons between male and female carrier samples were not conducted, they provided early quantitative evidence for a large discrepancy in neural involvement between male and female carriers, either symptomatic or sub-symptomatic. In contrast, in the present study, we conducted direct statistical comparison of the rate of progression in the three motor scale scores, and cognitive and psychiatric scores. Although there was significant progression in the ICARS and UPDRS scale scores over the 9–10 years period in the sample of apparently asymptomatic females, the rate of progression on the ICARS, especially the ICARS Gait domain, was demonstrably less than in the male sample. This differs from the negligible sex difference in the rate of progression for parkinsonian rest tremor on the UPDRS, and for the CRST. The latter result is not unpredictable, considering that this scale measures both kinetic (as in the ICARS) and parkinsonian (as a component of UPDRS) types of tremor.

Our findings concerning sex differences in the rate of progression are supported by the parallel cross-sectional study results comparing male and female carriers affected by FSD, group-matched for age. While there are large, highly significant differences between males and females on the ICARS, especially the ICARS Gait domain scores, this difference is negligible for the UPDRS score. Notably, the UPDRS was the only measure showing significant correlation with CGG repeat size in both male and female samples. Although the interpretation of these UPDRS findings is currently unclear, they throw new light on the relevance of the trajectory of parkinsonian features, as part of the FXTAS spectrum, to the premutation allele. Our results showing (highly) significant relationships between ICARS scores and CGG repeat size in females are also novel and intriguing. One earlier study reported significant relationships between CGG repeat size and the FXTAS Rating Scale scores in a relatively large sample of males, comprising both affected and non-affected premutation carriers, with a similar trend in the corresponding female sample (Leehey et al., 2008). We reported borderline significance of correlation between ICARS total and CGG in the male sample, but no significance with the gait or kinetic domains, possibly on account of extensive variability of these scores and the small sample size; a similar argument may apply to the CRST score, especially as they included the C domain of CRST, which is based on (highly subjective) reporting.

The pathogenesis of FSD involves an unstable “gain of function” mutation. The lower penetrance with respect to neural involvement in female compared with male premutation carriers is sometimes attributed to the effect of the second normal (and active) *FMR1* allele. However, the rates of progression in motor scores for tremor/ataxia observed in this study, being up to three times higher in males than in females, are greater than anticipated from the effect of random inactivation, which, in our study, was 0.57. This does not indicate skewed inactivation, and indeed, the level of mRNA in our female sample is almost exactly half of that in the male sample, which is as expected from random inactivation. However, the male-female differences in phenotype we observed were greater than would be expected from this, raising the possibility that other phenotype-modifying factors are operative in female carriers, even allowing for a discrepancy between blood and brain AR status (Tassone et al., 2004; Pretto et al., 2014a,b; Zhao et al., 2019). Earlier evidence for this view was provided by the absence of correlation between cognitive and/or neuropsychiatric scores and relevant molecular measures (CGG repeat, *FMR1* mRNA, and AR) in premutation females (Gossett et al., 2016; Jiraanont et al., 2017; Allen et al., 2020). Consistent with these data, there was no relationship between AR status and any of the phenotypic scores in a sample of 13 females from the “retrospective” sample included in this study (data not shown).

The present results provide further, more specific information on sex differences in motor dysfunction. Although these data are based on small samples, moderate to high effect sizes (Cohen, 1988) that we calculated for the differences in all motor scores give us sufficient confidence in the statistical assessment, and thus allow us to advance possible causes underlying these sex-related effects. The largest differences in males vs. females in both the progression rate in the longitudinal sample, and in the magnitude of motor impairment in the cross-sectional FXTAS sample, seen in the ICARS scores, suggested differential sex involvement of cerebellar vermal and anterior hemispheric structures. This sex difference appeared to be particularly evident for balance and gait dysfunction, reflected by ICARS (especially ICARS gait) subscores. Cognitive decline, affecting aspects of executive function, was evident only in male carriers. This is concordant with the motor findings, since these executive skills involve circuits that include the inferolateral cerebellar hemispheres (O'Halloran et al., 2012). Indeed, both the motor and the executive circuits' cortico-pontocerebellar afferents enter the cerebellum via the MCP. Given that the MCP sign, implying white matter degeneration, is very common in carrier males, but largely absent in carrier females, it is perhaps unsurprising that the sexes differ with respect to these two features of FSD. Unlike the tremor ataxia/scores, the parkinsonian score (UPDRS) showed similar values in both male and female FXTAS samples, as well as in the rate of progression. These results are consistent with the neuroradiological observation that, apart from the MCP sign, the pattern of white matter involvement is similar in the two sexes. For the illustration in Figure 4, we have shown, an example of a female who, despite an advanced form of FXTAS, did not show the MCP sign, whereas this sign was evident in a male with a milder form of FXTAS. Overall, we have encountered the MCP sign in all 24 FXTAS males, but in none of the females included in our cross-sectional analysis. Our clinical and neuroradiological data, showing disproportionately large gender differences in cerebellar manifestations, combined with small and/or insignificant gender differences in parkinsonian features, led to the conclusion that the cortico-cerebellar afferents (comprising the MCP) may be specifically protected in female carriers beyond the effect of the second normal (and active) allele. There is a possibility, though, that the activation ratio may be extremely biased in the cerebellar system, reflecting the wide variation of *FMR1* mRNA levels across different brain locations (Tassone et al., 2004). It is unlikely, however, since the elevation of this transcript in females is, in our study, half of that in males, and no evidence for any relationship of these levels with severity of cerebellar dysfunction. These results, though based on small samples, are interesting and indicative of the need for future studies based on larger samples and more direct approach to assess AR/mRNA relationships.

The most obvious limitation of this study is the smallness of our samples, implying that reliance on the *t*-test is potentially limited, mainly due to the elevated risk of Type II error. However, the moderate to large effect sizes regarding male-female differences in both progression rate and cross-sectional analysis of motor scores indicate that this risk is low for these comparisons. Although these effects are small to moderate for cognitive scores, the statistical results concerning motor scores provide critical evidence for this study's main hypothesis. Moreover, the effect of potentially large variances in some measures contributing to limited accuracy of the tests of significance has been reduced in this study by scoring, at each time point, being conducted by the same two neurologists. It may be noted that the confidence intervals (CI) determination were consistent with the results of the *t*-test for every measurement and in both progression and cross-sectional analysis. Another limitation relates to imperfect matching of male and female samples with respect to ascertainment, clinical categories, and interval between the repeat testing, though the latter was adjusted for in the analysis. However, it is practically impossible to achieve a perfect match between male and female carriers because, with rare exceptions, the trajectory and pattern of manifestations of the FSD, especially with respect to major features, is overtly different between the two genders. This difference may also have an impact on the mechanism of ascertainment, which may itself result in group differences. Moreover, our analysis makes the assumptions of linearity of both disease progression and assessment scales, whereas, in reality FXTAS may not necessarily progress evenly across time. We therefore corrected for these biases by adjusting the rate of progress data for the (T1) value representing baseline severity of involvement.

Using the term FSD to encompass a wide range of manifestations reminiscent of FXTAS, instead of classifying the affected individuals studied using the standard criteria for definitive, probable and possible categories (Hagerman and Hagerman, 2007), might be considered one of this study's limitations, especially since it restricts comparisons with other published results. However, our study has demonstrated that the transition from FSD to syndromic probable FXTAS is gradual; therefore, separating FXTAS from mild FSD may be artifactual. Furthermore, encompassing all the symptomatic carriers within the spectrum was determined by both small sample size and the lack of MRI results in some cases, and our reliance on the results of the motor rating scores. This approach is not only more relevant than the categorical one when employing these quantitative assessments, but it also reinforced the concept of a continuum of the effect of PM alleles with respect to neurological consequences.

However, despite these limitations, there is a consistent trend in the results from different aspects of the analysis., Our study reveal specific differences in the level and type of motor dysfunction between male and female carriers, and supports the hypothesis advanced in the study's aims: that subtle phenotypic changes, once initiated, continue to progress, but generally at a much slower and less uniform rate in female than in male PM carriers. These data, being suggestive of the existence of some sex-limited neuroprotective factors linked to the diminished cerebellar involvement in female carriers of the *FMR1* premutation allele, may indicate an avenue for future, more direct, neuropathological, and biochemical studies.

The search for possible protective factors in females should commence with investigation of cellular pathomechanisms already known to be involved in FXTAS. Mitochondrial dysfunction in FXTAS brains has been well documented (Giulivi et al., 2016; Alvarez-Mora et al., 2017), while the Rotterdam knock-in pre-CGG mouse model demonstrates raised cytoplasmic calcium levels (Robin et al., 2017). Neuronal calcium homeostasis is tightly controlled via regulation of mitochondrial and endoplasmic reticulum stores, and by several calcium-binding proteins including Calbindin D-28k, calretinin and parvalbumin (Bu et al., 2003). Calbindin levels are higher in the cerebellum and frontal cortex in female vs. male mice, and Calbindin D-28k null mice develop an ataxic phenotype (Barski et al., 2003). Calbindin levels are known to be controlled in part by estrogen receptor activation, although female estrogen receptor knock-out female XX mice still showed higher Calbindin levels than male XY receptor-knock-out mice (Abel et al., 2011). Therefore, Calbindin's effect on calcium regulation and the latter's interaction with mitochondrial function would appear to be a logical avenue for further exploration.

## Data Availability Statement

The raw data supporting the conclusions of this article will be made available by the authors, without undue reservation.

## Ethics Statement

The studies involving human participants were reviewed and approved by La Trobe University HREC Monash University HREC. The patients/participants provided their written informed consent to participate in this study.

## Author Contributions

DZL: conception, organization, and partial execution of research project, neurological assessments and motor scales scoring, review of statistical analysis, and co-writing (with ES) a manuscript. FT: conduct and interpretation of genetic molecular assays, review, and critique of manuscript. AA: contribution to cognitive testing and scoring, creating study database, execution of statistical analysis, contribution to review, and final editing of manuscript. NT: execution and description of the MR images in all study participants, review, and critique of manuscript. PS: conduct and interpretation of neuropsychological and psychiatric pathology assessments, organization, and partial execution of research project. DP: contribution to overall planning of this avenue of investigation and to interpretation of results in context with own data, and significant input to writing the Discussion. ES: conception and partial execution of research project, neurological assessments and motor scales scoring, neuropsychological assessments or supervision of assessments, and co-writing (with DZL) of manuscript. All authors: contributed to the article and approved the submitted version.

## Conflict of Interest

The authors declare that the research was conducted in the absence of any commercial or financial relationships that could be construed as a potential conflict of interest.
